# Safety and Efficacy of High Power Shorter Duration Ablation Guided by Ablation Index or Lesion Size Index in Atrial Fibrillation Ablation: A Systematic Review and Meta-Analysis

**DOI:** 10.1155/2021/5591590

**Published:** 2021-06-02

**Authors:** Xing Liu, Chun Gui, Weiming Wen, Yan He, Weiran Dai, Guoqiang Zhong

**Affiliations:** Department of Cardiology, The First Affiliated Hospital of Guangxi Medical University, Nanning 530000, Guangxi, China

## Abstract

**Background:**

High power shorter duration (HPSD) ablation may lead to safe and rapid lesion formation. However, the optimal radio frequency power to achieve the desired ablation index (AI) or lesion size index (LSI) is insubstantial. This analysis aimed to appraise the clinical safety and efficacy of HPSD guided by AI or LSI (HPSD-AI or LSI) in patients with atrial fibrillation (AF).

**Methods:**

The Medline, PubMed, Embase, Web of Science, and the Cochrane Library databases from inception to November 2020 were searched for studies comparing HPSD-AI or LSI and low power longer duration (LPLD) ablation.

**Results:**

Seven trials with 1013 patients were included in the analysis. The analyses verified that HPSD-AI or LSI revealed benefits of first-pass pulmonary vein isolation (PVI) (RR: 1.28; 95% CI: 1.05–1.56, P = 0.01) and acute pulmonary vein reconnection (PVR) (RR: 0.65; 95% CI: 0.48–0.88, P = 0.005) compared with LPLD. HPSD-AI or LSI showed higher freedom from atrial tachyarrhythmia (AT) (RR = 1.32, 95% CI: 1.14–1.53, P = 0.0002) in the subgroup analysis of studies with PVI ± (with or without additional ablation beyond PVI). HPSD-AI or LSI could short procedural time (WMD: −22.81; 95% CI, −35.03 to −10.60, P = 0.0003), ablation time (WMD: −10.80; 95% CI: −13.14 to −8.46, P < .00001), and fluoroscopy time (WMD: −7.71; 95% CI: −13.71 to −1.71, P = 0.01). Major complications and esophageal lesion in HPSD-AI or LSI group were no more than LDLP group (RR: 0.58; 95% CI: 0.20–1.69, P = 0.32) and (RR: 0.84; 95% CI: 0.43–1.61, P = 0.59).

**Conclusions:**

HPSD-AI or LSI was efficient for treating AF with shorting procedural, ablation, and fluoroscopy time, higher first-pass PVI, and reducing acute PVR and may increase freedom from AT for patients with additional ablation beyond PVI compared with LPLD. Moreover, complications and esophageal lesion were low and no different between two groups.

## 1. Introduction

Compared to medical therapies alone, catheter ablation has been identified as an effective treatment for atrial fibrillation (AF), and quality of life of patients was significantly improved [1]. Pulmonary vein isolation (PVI) acted as the cornerstone for radiofrequency ablation of AF. The efficacy of radiofrequency catheter ablation (RFCA) is related to transmural, continuous, and cellular necrosis [2]. The conventional ablation therapy is mainly low power longer duration (LPLD). High power primarily increases the effect of resistive heating, while ablation duration produces conductive heating. Irreversible myocardial tissue damages with cellular necrosis are rapidly induced by resistive heating, whereas conductive heating passively stretches into deeper tissue layers, resulting in potential reversible tissue injuries. Moreover, it is quite difficult to retain catheter stability in a beating heart for a long time, and tissue edema caused by prolonged ablation hinders effective ablation [2], leading to the rate of pulmonary vein reconnection (PVR) that maintains frequently with LPLD. Simultaneously, LPLD ablation may generate damage depth excessively, thus increasing the risk of adjacent tissue damage, especially esophageal thermal injury (ETI) [3].

High power shorter duration (HPSD), as a novel ablation strategy, has been applied in AF treatment [4]. HPSD was safe and efficient for treating AF with shorting procedural and ablation time and higher first-pass pulmonary vein isolation (PVI), but it did not significantly reduce recurrence of atrial tachyarrhythmia (AT) compared with LPLD [5, 6]. Recurrent AT after PVI is generally associated with PVR, and gaps in the circumferential pulmonary veins (PVs) isolation lines are accompanied by increased recurrence of AF [7]. A weighted proprietary formula such as ablation index (AI) or lesion size index (LSI) incorporated with contact force (CF), radiofrequency (RF), application time, and power was reported to be beneficial to produce durable ablation lesion and to minimize AF recurrence following ablation [8, 9]. Recently, high-powered ablation guided by AI or LSI (HPSD-AI or LSI) was safe and procedural efficiency reduced with recurrence of AT [10, 11]. However, results of arrhythmia-related outcomes are contradictory and inconclusive [12, 13]. Therefore, we conducted systematic reviews and meta-analyses to evaluate the efficacy and safety of HPSD-AI or LSI compared with LPLD in treating AF.

## 2. Methods

### 2.1. Search Strategy

An all-round search was searched in the Medline, PubMed, Embase, Web of Science, and the Cochrane Library databases from inception up to November 2020 by two reviewers (XL and CG) independently. Articles in non-English languages were excluded. The following search strategy was applied to search PubMed, and we adapted it for the other databases: (“High-power” [Title/Abstract] OR ““HPSD” [Title/Abstract]) AND (“AF” [Title/Abstract] OR ((“atrial” [Title/Abstract] OR “atrium” [Title/Abstract] OR “auricular” [Title/Abstract]) AND (“fibrillation^*∗*^” [Title/Abstract] OR “arrhythmia^*∗*^ [Title/Abstract] OR “flutter^*∗*^” [Title/Abstract])) OR (“Atrial Fibrillation” [MeSH Terms] OR “Atrial Flutter” [MeSH Terms])).

### 2.2. Inclusion and Exclusion Criteria

Two investigators (XL and WW) filtrated and identified research studies that fulfilled the following inclusion criteria: (1) full text studies of controlled experiments about HPSD-AI or LSI versus LPLD; LPLD: power ≤ 35 W, with a longer ablation duration of 10 to 30s per site; HPSD-AI or LSI: power ≥40 W, duration ≤ 10 s in ablation or less than LPLD group, with LSI ≥ 4 or AI ≥ 350 in sites on the LA posterior wall and LSI ≥ 5 or AI ≥ 400 in others; (2) patients with AF who consented radiofrequency ablation; (3) without a AF ablation history; (4) PVI applied using the contact force catheter; and (5) studies wanted to provide some dependable information with first-pass PVI, regarding procedure outcomes, acute PVR, either recurrence rates of AT including AF and atrial flutter, and complications in both groups. The exclusion criteria were as follows: (1) ablation used the noncontact force catheter; (2) studies enrolled less than 10 patients; and (3) animal studies, conference abstracts, case reports, review articles, editorials, or non-English language articles.

### 2.3. Quality Assessment

The study quality was evaluated by two investigators (WW and YH) using the Newcastle–Ottawa scale (NOS) for nonrandomized studies. And a star system (0–9) was used to judge studies. A research with NOS ≥7 was judged to be a study of good quality [14]. The quality of randomized controlled trials (RCTs) was evaluated by the Cochrane Collaboration tool for assessing risk of bias [15].

### 2.4. Data Extraction

Data were extracted using standardized protocol and reporting forms, including name of the first author, year of publication, country of origin, sample size, baseline characteristics (age, gender, left atrial diameter, and CHA2DS2-VASc), ablation strategy, ablation procedure details, AF type, ablation catheter type, the mapping system, freedom from AT, and procedure-related complications. The sample mean and standard deviation from commonly reported quantiles are estimated [16]. This data extraction process was performed independently by two investigators (XL and WD). Discrepancies between them were resolved by a third reviewer (CG).

### 2.5. Statistical Analysis

Dichotomous variables and outcome endpoints were reported as a risk ratio (RR) with 95% confidence intervals (CIs). The continuous variables were analyzed using weighted mean differences (WMD) or standard mean differences (SMD). The between-study heterogeneity was reflected by *I*^2^ > 50%, with *P* < 0.05 deemed statistically significant. In cases of heterogeneity, random-effects models were used; otherwise (*I*^2^ ≤ 50%), fixed-effects models were preferentially used. In cases of statistical heterogeneity, subgroup analysis or sensitivity analyses were used. Sensitivity analysis was performed to determine the consistency of the overall effect estimate. When the pooled analysis still yielded significant heterogeneity, descriptive analysis was used. All *P* values were two-tailed with a statistical significance set at 0.05. Publication bias was assessed by using the funnel plots. The statistical analysis was performed using the Revman5.4 software.

## 3. Results

### 3.1. Study and Data Selection

**Figure 1:**
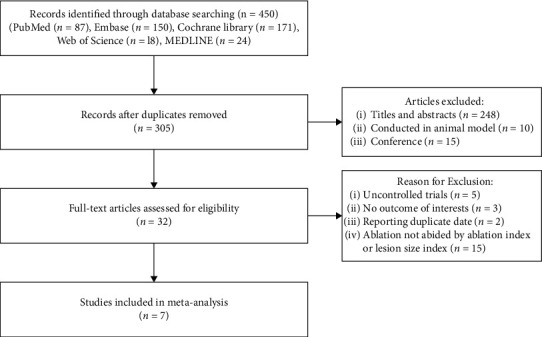
The flowchart of the literature search strategy.

The results of the detailed search process are shown in Figure 1. Initially, 450 potentially relevant studies were yielded in our search strategy, of which 145 were duplicates and 248 were excluded after title and abstract review and abstracts. Of the remaining, 25 studies were excluded as topics were conducted in animals and conference, leaving a total of 32 studies for reading the full text. At this stage, further 25 studies were excluded after a detailed assessment of the full text due to the following: 5, uncontrolled trials; 3, no outcome of interests; 2, reporting duplicate date; and 15, ablation not abided by AI or LSI. No additional studies were added through manual search. Thus, 7 studies were finally selected in this meta-analysis [10–13, 17–19].

### 3.2. Study Characteristics and Quality Assessment of Included Studies

The characteristics of the included trials and ablation settings are summarized in Tables 1 and 2. A total of 1013 patients (409 patients underwent HPSD-AI or LSI strategy and 526 patients underwent LPLD strategy) were included in the analysis. There were four prospective cohort trials and two retrospective cohort trials and one RCT. There is no consensus about the power and AI or LSI for HPSD; in our study, energy levels at or above 40 W are considered as high power. The target ablation lesion index was reached: LSI ≥ 4 or AI ≥ 350 in sites on the LA posterior wall and LSI ≥ 5 or AI ≥ 400 in others [10–13, 17–19]. In the case of esophageal heating >38.5° or 39°, the AI target of the entire posterior ostium of that vein was lowered to ≥300 [17, 18]. Even the target AI was set at 260 on the esophagus in each ablation point [12]. The only RCT of energy difference between HPSD and LPLD was reflected only in the posterior wall ablation conducted by Leo et al. [11]. Meanwhile, we divided the study into two groups according to the difference of LSI in sites on the LA posterior wall (group 1, LSI of 4, group 2, LSI of 5). One trail by Okamatsu et al. [12] including three groups (low power, medium power, and high power) and medium-power group (≥40 W) was enrolled into HPSD-AI or LSI group according to the inclusion criteria of our study. Another trial by Castrejón-Castrejón et al. [18] containing the subgroup of power of 60 W was excluded because their ablation was not guided by LSI. In two studies [13, 17], PVI alone was performed except for cavotricuspid isthmus ablation because a typical atrial flutter was documented before or during the operation. One study [13] included only patients with paroxysmal atrial fibrillation.

Quality assessment of included studies is given in Table 3. None of the included studies was of poor quality.

### 3.3. First-Round Isolation Rate

**Figure 2:**
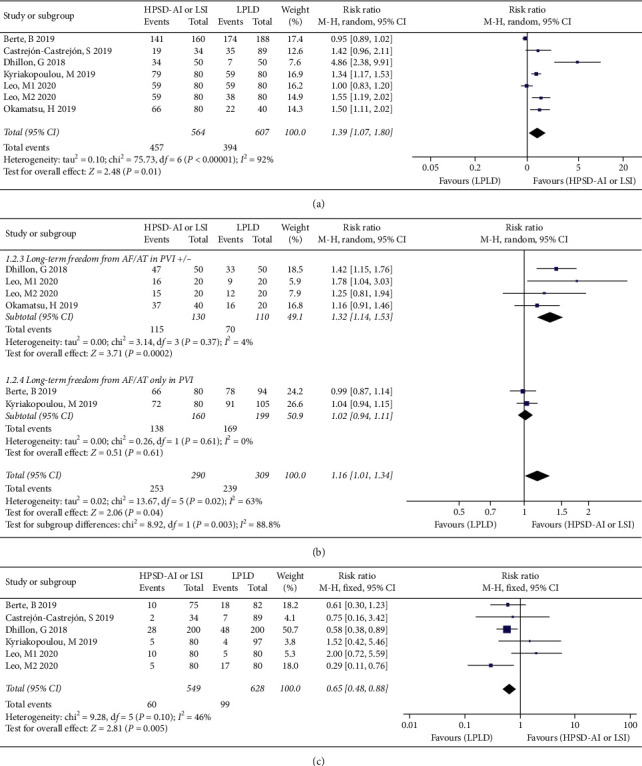
Forest plot displaying the efficacy outcomes in the HPSD-AI or LSI group compared to the LPLD group. (a) First-pass PVI, (b) long-term freedom from AF/AT, and (c) acute PVR.

6 studies [10–13, 17, 18] reported the first-round isolation rate. The first-round isolation rate of PVs in the HPSD-AI or LSI group was significantly higher than in the LDLP group (RR: 1.28; 95% CI: 1.05–1.56, *I*^2^ = 92%, *P*=0.01) (Figure 2). Considering the high heterogeneity, the random-effects model was used for analysis. By sensitivity analysis by removing any individual study, the results did not change, indicating that the results were stable.

### 3.4. Acute PV Reconnection (APR) Rate

The APR rate was reported in 5 included studies [10, 11, 13, 17, 18] and the heterogeneity was low (*I*^2^ = 46%). The APR rate in the HPSD-AI or LSI group was significantly lower compared with the LDLP group (RR: 0.65; 95% CI: 0.48–0.88, *P*=0.005) (Figure 2).

### 3.5. Long-Term Freedom from AF/AT

More than 6 months follow-up outcomes were summarized from 5 studies [10–13, 17], and the heterogeneity was moderate (*I*^2^ = 63%). More than 6 months success rate in the HPSD-AI or LSI group was higher than in the LDLP group (RR = 1.16, 95% CI: 1.01–1.34, *P*=0.04) (Figure 2). Subgroup analysis was performed according to ablation strategies to analyze the source of high heterogeneity. 3 studies [10–12] PVI ± (with or without line, Box isolation or complex fractionated atrial electrogram ablation) were included, and the heterogeneity was very low (*I*^2^ = 4%). Long-term freedom from AF/AT 6 months or later after the AF ablation in the HPSD-AI or LSI group was also significantly higher than in the LDLP group (RR = 1.32, 95% CI: 1.14−1.53, *P*=0.0002) (Figure 2). 2 studies [13, 17] with only PVI except a typical atrial flutter performed by cavotricuspid isthmus ablation was included, and the heterogeneity was very low (*I*^2^ = 0%). There was no significant difference in terms of recurrence of AF/AT in two groups (RR = 1.02, 95% CI: 0.94−1.11, *P*=0.61). The results of the sensitivity analysis were not altered by the deletion of any individual studies from the analysis.

### 3.6. Procedure Efficiency

**Figure 3:**
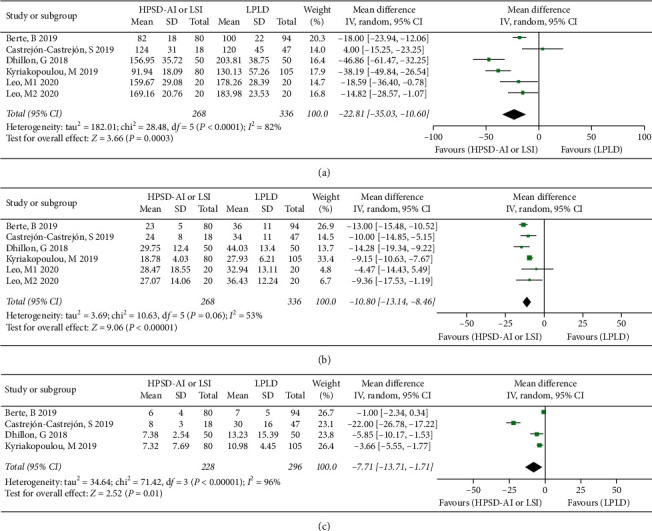
Forest plot displaying procedural efficiency. (a) Procedure duration, (b) radiofrequency duration, and (c) fluoroscope duration.

Results including procedure, ablation, and fluoroscopy times were available in 5, 5, and 4 of the studies, respectively [10, 11, 13, 17, 18]. There was a significant reduction in the procedure time (WMD: −22.81; 95% CI: −35.03 to −10.60, *I*^2^ = 82%, *P*=0.0003), ablation time (WMD: −10.80; 95% CI: −13.14 to −8.46, *I*^2^ = 53%, *P* < .00001), and fluoroscopy time (WMD: −7.71; 95% CI: −13.71 to −1.71, *I*^2^ = 95%, *P*=0.01) (Figure 3). Considering the high heterogeneity, the random-effects model was used for all analyses. The sensitivity analysis showed the results were not driven by any single study.

### 3.7. Procedural Complications

**Figure 4:**
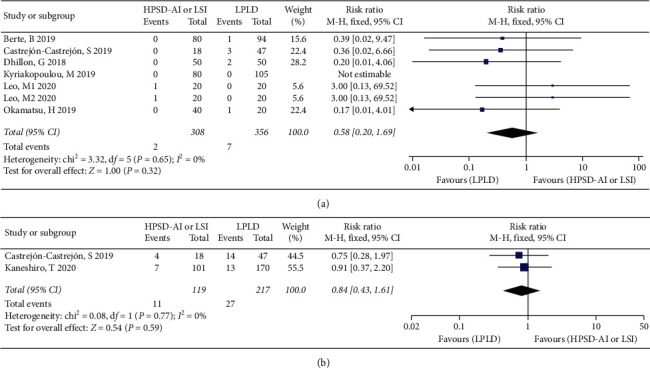
Forest plot displaying risk estimates of the primary safety outcome. (a) Complication rate and (b) esophageal lesion rate.

Procedural complications mainly referred to aterioesophageal fistula, pericardial effusion/cardiac tamponade, and stroke were reported in 6 studies [10–13, 17, 18]. There were no significant differences in procedural complications between the two groups (RR: 0.58; 95% CI: 0.20−1.69, *I*^2^ = 0%, *P*=0.32) (Figure 4). Esophageal lesions were evaluated by esophagogastroduodenoscopy in two trials [18, 19]. There were no significant differences in esophageal lesions between the two groups (RR: 0.84; 95% CI: 0.43–1.61, *I*^2^ = 0%, *P*=0.59) (Figure 4). The fixed-effects model was used for analyses because of the very low heterogeneity. By removing any individual studies for sensitivity analysis, there was no significant change in the point estimate or CI in the results.

### 3.8. Publication Bias

We intended to investigate potential publication bias by funnel plots. However, since there were only as many as seven studies in our main analysis, the number was insufficient to reject the hypothesis of no funnel plot asymmetry. So we did not perform a funnel plot [20, 21].

## 4. Discussion

### 4.1. Major Findings

This study represented the first systematic review and meta-analysis on the comparison between HPSD-AI or LSI ablation and LPLD in patients with AF. The main findings were as follows: (1) HPSD-AI or LSI ablation showed higher first-round isolation rate and lower APR rate compared with LPLD, (2) The HPSD-AI or LSI group had a higher freedom from AF/AT 6 months or later after AF ablation than the LDLP group. There was a similarity between two groups in freedom from AF/AT among patients undergoing only PVI under the subgroup analysis of ablation strategy, but freedom from AF/AT rate was also significantly higher than in the LDLP group in the PVI ± subgroup, (3) HPSD-AI or LSI strategy could meaningfully shorter procedural, ablation, and fluoroscopy time compared with the LPLD, and (4) major complications and esophageal lesions were similar between two groups.

### 4.2. Clinical Efficacy

AI or LSI incorporated CF, RF application time, and power into a weighted proprietary formula and experimental research has revealed that lesion depth can be predicted accurately by the AI formula and power-made more contributions than CF at the initial time of ablation [8, 22]. Meanwhile, recent clinical trials targeting AI values of 550 at the anterior wall of left atrial (LA) and 400 at the LA posterior wall were related to high single operation success rate and low rate of PVR [9]. As a novel energy delivery strategy, HPSD was used to optimize LPLD. It is well known that catheter instability or poor contact may induce incomplete lesions and tissue edema during radio frequency delivery. In turn, it is difficult to achieve transmural injuries with further radiofrequency applications, resulting in conduction gaps and PV reconnections. On the contrary, HPSD can improve the stability of catheter in a short time and increase the injure area through predominant resistive heating [2]. Previous clinical studies using “uncontrolled” high power ablation for PVI showed a meaningfully shorter fluoroscopic time, procedural time, higher rate of first-pass PVI, and similar freedom from AF/AT rate [5, 23], in which results were consistent with our meta-analysis except for the last one. However, an observational study found that HPSD ablation was related to a higher risk of atrial flutter and a potential surrogate for incomplete sets/lines [24].

Recently, the study by Chen et al. [25] demonstrated that the initial 6-month follow-up showed 48 (96%) patients were free from clinical AF/AT recurrence by AI-guided 50 W ablation. Therefore, combining the superiority of the high-power ablation abided by the AI or LSI may better balance the procedural efficacy and safety. Winkle et al. [26] targeted LSI values of 5.5–6 for LA ablation at 50 W and reported a low complication rate and single procedure freedom from AF of 83% for paroxysmal AF and 72% for persistent AF at 2 years. By comparing HPSD-AI or LSI and LPLD studies, our analysis gets the same results as Chen et al. [25] who reported on their study that HPSD-AI or LSI was associated with increased more than 6-month freedom from AF/AT compared to LPLD ablation. Considering the high heterogeneity of the results, the subgroup analysis of different ablation strategies showed good homogeneity. The same conclusion was reached in the PVI ± subgroup, while freedom from AF/AT rate of HPSD-AI or LSI was not more than LDLP among patients with undergoing only PVI, indicating that HPSD-AI or LSI may increase freedom from AT for patients with additional ablation beyond PVI compared with LPLD. The possible reason is that HPSD-AI or LSI is superior than LPLD in improving the success rate of additional ablation beyond PVI and reducing the incidence of associated arrhythmias after radiofrequency ablation of AF. What merits our attention is that high power ablation can achieve the AI or LSI target in a shorter time. Importantly, clinical studies that used a significantly higher power of 70 W for 5–7 s and very higher power of 90 W for 4s have shown that therapeutic effects can be achieved, but AI or LSI has become irrelevant [27, 28]. AI is generally used as the local lesion endpoint only when ≤50 W because a reliable local lesion endpoint cannot be determined especially when very high power is used in a few seconds. This means that the operator needs to latently terminate the ablation lesion before the AI or LSI value is made visible. Otherwise, it would add the risk of overtreatment and potential complications. Therefore, large sample randomized controlled studies are needed to confirm how high the power is.

### 4.3. Procedural Efficiency

In terms of procedural efficiency, the pooled analysis revealed that HPSD-AI or LSI ablation can extraordinarily reduce the RF ablation time, procedure time, and fluoroscopy time compared to LDLP, which are consistent with a meta-analysis about comparison of HPSD and LDLP ablation [6], thus limiting patient exposure to intravenous fluids that could be beneficial in reducing the risk of postablation cognitive dysfunction [29]. Meanwhile, shorter radiation duration directly benefits the patient, operator, and supporting staff. In contrast, longer ablation time and procedure time in the LPLD group may increase surgical complications. Due to a reduction in RF time because of HPSD guided by AI or LSI formula, the procedure time obviously shortens. And shorter ablation time was because of the shorter time required for lesion creation, higher first-pass PVI, and fewer acute PV reconnections than those of LPLD, which are consistent with our analysis results.

### 4.4. Safety

The most concern was about the safety issue of HPSD-AI or LSI ablation. Under the premise of achieving fulfilling procedural efficiency and efficacy outcomes, HPSD-AI or LSI of major complications and esophageal lesions were similar with the LPLD group in our meta-analysis, which were consistent across all included study that reported this result [10–13, 17–19]. But, in the 5 included studies [10, 12, 13, 17, 18], cases of aterioesophageal fistula or cardiac tamponade were not observed in the HPSD-AI group or the LSI group. One case of cardiac tamponade occurring in the HPSD-LSI group reported by Leo et al. [11] was presumably due to inadvertent transseptal puncture via the transverse sinus instead of ablation. However, three (7%) patients in the LPLD group developed cardiac tamponade and required urgent pericardiocentesis, and one of them occurred following an audible steam pop during cavotricuspid isthmus ablation [18]. Importantly, whether high power can reduce esophageal damage is our concerned question. It is all known that left atrial-esophagus fistula is a fatal complication associated with PVI. HPSD approach can adjust the relationship between resistive and conductive heating, avoiding potential colleterial damage to adjacent structures such the esophagus [2]. Recent clinical studies [19] have shown that the incidence of ETI was significantly higher in the HPSD group compared to the LPLD group (37% vs. 22%, *P*=0.011), but the prevalence of esophageal lesions did not differ between the groups (7% vs. 8%). The use of the HPSD setting could avoid deeper thermal injuries that reach the esophageal mucosal layer because it was a strong predictor of ETI. All esophageal lesions inspected by gastroscopy in the HPSD group were mild erythema, and the esophageal lesions in the LPLD group showed ulceration, which also suggests that thermal injury could not reach the esophageal mucosal layer deeply when using the HPSD setting. Consistently, Wolf M et al. also reported low rate of esophageal lesions (1.2%, more than 7 days) following the AI-guided PVI [30]. Animal experiment on the pig model found that HPSD ablation can significantly reduce the lesion volume and cause less damage to the esophagus when AI is taken as a predefined target for different power settings [31]. Thus, a large sample randomized controlled study may conclude that HPSD-AI or LSI may cause less esophageal damage than LPLD.

## 5. Limitation

This meta-analysis has some limitations. First, there were variations in the high power definition and AI or LSI setting between the included studies as we analyzed. Second, there were different operator experiences, types of catheters, irrigation fluid delivery rate, and ablation strategy, all of which led to otherness in lesion formation. Data, which were extracted from the included studies, were not adjusted for these. Third, the included studies did not compare HPSD-AI or LSI with LPLD in patients with paroxysmal and persistent AF separately. Fourth, only seven studies with small sample size were included in our meta-analysis, and only one of them was RCT. Thus, more well-designed and large-scale RCTs with large sample size and longer term follow-up are demanded to validate the safety and efficiency of HPSD-AI or LSI strategy. Fifth, most included studies did not monitor esophageal temperature and perform gastroscopy, resulting in limitations in assessing esophageal damage.

## 6. Conclusions

Our systematic review and meta-analysis showed that HPSD-AI or LSI was effective method for AF ablation. Compared with the LPLD approach, it had some obvious advantages, including shorter procedure time, ablation time, and fluoroscopy time. In addition, HPSD-AI or LSI approach had higher first-pass PVI and lower acute PV reconnection and may increase freedom from AT for patients with additional ablation beyond PVI. Moreover, complications and esophageal lesion were low and there was no difference between the two groups. Further randomized multicenter studies with larger sample sizes and longer term follow-up are necessary to confirm the safety of HPSD-AI or LSI.

## Figures and Tables

**Table 1:** Baseline characteristics of included studies.

Study	Country	Study type	Treatment group	Patients (*n*)	Follow (month)	Age (years)	Male (%)	BMI	DM (%)	PAF (%)	LVEF (%)	LAD (mm)	CHA2DS2-VASc
Leo et al. [11]	United Kingdom	Randomized controlled trial	HPSD-LSI 1	20	29	60.1 ± 9.4	60	27.3 ± 5	N/A	40	60.0 ± 9.2	41.4 ± 6.5	1 (IQR: 1–3)
			LPLD-LSI 1	20	29	58.9 ± 9.2	95	30.8 ± 4.6	N/A	45	60.0 ± 11.5	43.0 ± 6	1 (IQR: 0–2)
			HPSD-LSI 2	20	29	61.3 ± 9.6	70	28.8 ± 4.9	N/A	40	57.9 ± 6.4	43.7 ± 9.3	2 (IQR: 0–4)
			LPLD-LSI 2	20	29	55.7 ± 10	70	28 ± 4.85	N/A	30	60.0 ± 10.2	42.4 ± 7.7	1 (IQR: 1-2)

Kaneshiro et al. [19]	Japan	Retrospective cohort trial	HPSD-AI	101	N/A	63 ± 10	76	N/A	N/A	66	N/A	40.8 ± 6.3	N/A
			LPLD	170	N/A	61 ± 10	81	N/A	N/A	79	N/A	38.8 ± 6.5	N/A

Berte et al. [17]	Switzerland	Prospective cohort trial	HPSD-AI	80	6	62 ± 9	72	N/A	40	81	58 ± 8	N/A	N/A
			LPLD-AI	94	6	63 ± 9	71	N/A	31	79	59 ± 11	N/A	N/A
Okamatsu et al. [12]	Japan	Prospective cohort trial	HPSD-AI1	20	6	65 ± 10	65	N/A	25	65	65 (IQR: 60–71)	40 ± 6	2 (IQR: 1–3)
			LPLD-AI	20	6	68 ± 8	75	N/A	5	80	64 (IQR: 60–67)	39 ± 6	2 (IQR: 1‐2)
			HPSD-AI2	20	6	64 ± 8	55	N/A	19	75	64 (IQR: 59–71)	40 ± 5	2 (IQR: 1–3)

Castrejon-Castrejon et al. [18]	Spain	Prospective cohort trial	HPSD50w-AI or LSI	18	N/A	N/A	N/A	N/A	N/A	N/A	N/A	N/A	N/A
			HPSD60w	30	N/A	N/A	N/A	N/A	N/A	N/A	N/A	N/A	N/A
			LPLD	47	N/A	N/A	60	29 ± 5	N/A	64	56 ± 11	N/A	N/A

Kyriakopoulou et al. [13]	Belgium	Retrospective cohort trial	HPSD-AI	80	12	67 (IQR: 58–73)	59	28 ± 5	N/A	100	N/A	43 ± 8	2 (IQR: 1–3)
			LPLD-AI	105	12	64 (IQR: 56–69)	62	27 ± 4	N/A	100	N/A	44 ± 6	2 (IQR: 1‐2)

Dhillon et al. [10]	United Kingdom	Prospective cohort trial	HPSD-AI	50	12	N/A	N/A	N/A	N/A	N/A	N/A	N/A	N/A
			LPLD	50	12	N/A	N/A	N/A	N/A	N/A	N/A	N/A	N/A

Values are mean ± SD, median (interquartile range), or *n*%; N/A, not available; AI, ablation index; BMI, body mass index; CHA2DS2-VASc, cardiac failure or dysfunction, hypertension, age ≥75 (doubled), diabetes, stroke (doubled)-vascular disease, age (65–74), and sex category (female); DM, diabetes mellitus; HPSD, high power and short duration; IQR, interquartile range; LAD, left atrial diameter; LPLD, low power longer duration; LSI, lesion size index; LVEF, left ventricular ejection fraction; PAF, paroxysmal atrial fibrillation.

**Table 2:** Procedural characteristics.

Study	Treatment group	CF sensing catheter/Agilis sheath	STSF catheter/Agilis sheath	Mapping system	Anterior/Posterior wall power	Local lesion endpoint	Ablation strategy
Leo et al. [11]	HPSD-LSI 1	+/+	−	EnSite	40 W	Target LSI of 5.5–6 at the LA anterior wall and 4 at the posterior wall	PVI ± line
	LPLD-LSI 1	+/+	−	EnSite	40 W/20 W	Target LSI of 5.5–6 at the LA anterior wall and 4 at the posterior wall	PVI ± line
	HPSD-LSI 2	+/+	−	EnSite	40 W	Target LSI of 5.5–6 at the LA anterior wall and 5 at the posterior wall	PVI ± line
	LPLD-LSI 2	+/+	−	EnSite	40 W/20 W	Target LSI of 5.5–6 at the LA anterior wall and 5 at the posterior wall	PVI ± line

Kaneshiro et al. [19]	HPSD-AI	−	+/−	CARTO	45–50 W	Target AI of 400 at the LA posterior wall	PVI ± line
	LPLD	+−/−	+−/−	CARTO	20–30 W	Duration at 10–30 s, CF 20–30 g	PVI ± line

Berte et al. [17]	HPSD-AI	−	+/+	CARTO	45 W/35 W	Target AI of 550 at the LA anterior wall and 300–400 at the posterior wall	PVI
	LPLD-AI	−	+/+	CARTO	35 W/25 W	Target AI of 550 at the LA anterior wall and 300–400 at the posterior wall	PVI

Okamatsu et al. [12]	HPSD-AI1	−	+/+−	CARTO	50 W/30–40 W	Target AI of 400 at the LA anterior wall and 260–360 at the posterior wall	PVI ± line ± box isolation ± CFAE
	LPLD-AI	−	+/+−	CARTO	30 W/20 W	Target AI of 400 at the LA anterior wall and 260–360 at the posterior wall	PVI ± line ± box isolation ± CFAE
	HPSD-AI2	−	+/+−	CARTO	40 W/30 W	Target AI of 400 at the LA anterior wall and 260–360 at the posterior wall	PVI ± line ± box isolation ± CFAE

Castrejon-Castrejon et al. [18]	HPSD50w-AI or LSI	+/+−	−	CARTO/ EnSite	50 W	LSI≥ 5, AI≥ 350 at the LA posterior wall and ≥450 in others	PVI ± line
	HPSD60w	+/+−	−	CARTO/EnSite	60 W	2–7 s	PVI ± line
	LPLD	+/+−	−	CARTO/EnSite	30 W/20–30 W	30–60 s	PVI ± line

Kyriakopoulou et al. [13]	HPSD-AI	+/−	−	CARTO	40 W	Target AI of 550 at the LA anterior wall and 300–400 at the posterior wall	PVI
	LPLD-AI	+/−	−	CARTO	35 W	Target AI of 550 at the LA anterior wall and 300–400 at the posterior wall	PVI

Dhillon et al. [10]	HPSD-AI	−	+/−	CARTO	40 W/30 W	Target AI of 450 at the LA anterior wall and 350 at the posterior wall	PVI ± line
	LPLD	+/−	−	CARTO	30 W/25 W	CF 20–30 g	PVI ± line

AI, ablation index; CF, contact force; CFAE, complex fractionated atrial electrogram; CTI, cava-tricuspid isthmus isolation; HPSD, high power shorter duration; LA, left atrial; LPLD, low power longer duration; LSI, lesion size index; PVI, pulmonary vein isolation; STSF, ThermoCool SmartTouch Surround Flow; SVCI, superior vena cava isolation.

**Table 3:** Quality assessment of the included studies according to the Newcastle–Ottawa scale or Cochrane Collaboration tool for assessing risk of bias.

Study	Representativeness of the exposed cohort	Selection of the nonexposed cohort	Ascertainment of exposure	Demonstration that outcome of interest was not present at start of the study	Comparability of cohorts on the basis of the design or analysis	Assessment of outcome	Was follow‐up long enough for outcomes to occur	Adequacy of follow-up of cohorts	Total stars
Kaneshiro et al. [19]	^*∗*^	^*∗*^	—	^*∗*^	^*∗*^	^*∗*^	—		6
Berte et al. [17]	^*∗*^	^*∗*^	^*∗*^	^*∗*^	^*∗*^	^*∗*^	^*∗*^	^*∗*^	9
Okamatsu et al. [12]	^*∗*^	^*∗*^	^*∗*^	^*∗*^	^*∗*^	^*∗*^	^*∗*^	^*∗*^	9
Castrejon-Castrejon et al. [18]	^*∗*^	^*∗*^	^*∗*^	—	^*∗*^	^*∗*^	^*∗*^		7
Kyriakopoulou et al. [13]	^*∗*^	^*∗*^	—	^*∗*^	^*∗*^	^*∗*^	^*∗*^	^*∗*^	8
Dhillon et al. [10]	^*∗*^	^*∗*^	—	^*∗*^	^*∗*^	^*∗*^	^*∗*^	^*∗*^	8
Study	Random sequence generation	Allocation concealment	Blinding of participants and personnel	Blinding of outcome assessment	Incomplete outcome data	Selective reporting	Other bias		
Leo et al. [11]	Low risk	Low risk	Low risk	Low risk	Low risk	Unclear risk	Unclear risk		

## Data Availability

The data used to support the findings of this study are included within the article.
